# Evaluating the Frequency of *aac(6′)-IIa*, *ant(2″)-I*, *intl1*, and *intl2* Genes in Aminoglycosides Resistant *Klebsiella pneumoniae* Isolates Obtained from Hospitalized Patients in Yazd, Iran

**Published:** 2018

**Authors:** Hesam Mokhtari, Gilda Eslami, Hengameh Zandi, Amin Dehghan-Banadkouki, Mahmood Vakili

**Affiliations:** 1. International Campus, Shahid Sadoughi University of Medical Sciences, Yazd, Iran; 2. Research Center for Food Hygiene and Safety, Shahid Sadoughi University of Medical Sciences, Yazd, Iran; 3. Department of Parasitology and Mycology, Faculty of Medicine, Shahid Sadoughi University of Medical Sciences, Yazd, Iran; 4. Department of Microbiology, Faculty of Medicine, Shahid Sadoughi University of Medical Sciences, Yazd, Iran; 5. Department of Pathobiology, Faculty of Public Health, Tehran University of Medical Sciences, Tehran, Iran; 6. Department of Public Medicine, Faculty of Medicine, Shahid Sadoughi University of Medical Sciences, Yazd, Iran

**Keywords:** Aminoglycosides, Drug resistance, Integrons, *Klebsiella pneumoniae*, Microbial

## Abstract

**Background:**

*Klebsiella pneumoniae (K. pneumoniae)* is an opportunistic pathogen that could be resistant to many antimicrobial agents. Resistance genes can be carried among gram-negative bacteria by integrons. Enzymatic inactivation is the most important mechanism of resistance to aminoglycosides. In this study, the frequencies of two important resistance gene *aac(6′)-II*a and *ant(2″)-I,* and genes coding integrase I and II, in *K. pneumoniae* isolates resistant to aminoglycosides were evaluated.

**Methods:**

In this cross-sectional study, an attempt was made to assess the antibiotic susceptibility of 130 *K. pneumoniae* isolates obtained from different samples of patients hospitalized in training hospitals of Yazd evaluated by disk diffusion method. The frequencies of *aac(6′)-II*a, *ant(2″)-I, intl1*, and *intl2* genes were determined by PCR method. Data were analyzed by chi-square method using SPSS software (Ver. 16).

**Results:**

our results showed that resistance to gentamicin, tobramycin, kanamycin, and amikacin were 34.6, 33.8, 43.8, and 14.6%, respectively. The frequencies of *aac (6′)-II*a, *ant(2″)-I, intl1*, and *intl2* genes were 44.6, 27.7, 90, and 0%, respectively.

**Conclusion:**

This study showed there are high frequencies of genes coding aminoglycosides resistance in *K. pneumoniae* isolates. Hence, it is very important to monitor and inhibit the spread of antibiotic resistance genes.

## Introduction

*Klebsiella pneumoniae* (*K. pneumoniae*) is an opportunistic pathogen that mainly causes nosocomial infections^1^. This bacterium can cause serious illnesses, such as septicemia, *pneumoniae*, bacteremia, urinary tract and soft tissue infections. Emergence of antibiotic resistant strains challenges the efficacy of antibiotic drugs; and the number of antibiotic resistant strains is increasing^2^.

Aminoglycosides are a group of antibiotics that are very effective against a broad spectrum of gram-positive and gram-negative aerobic bacteria. These antibiotics are the most widely used drugs for treatment of infections caused by facultative anerobic gram-negative rods^3^. Despite widespread usage and clinical success of aminoglycosides, administration of these anti-biotics for controlling bacterial infections has been challenged by emergence of resistant strains. Inactivation by a wide range of enzymes known as Aminoglycosides Modifying Enzymes (AMEs), is the most common mechanism of inactivation of aminoglycosides.

AMEs inactivate aminoglycosides through several molecular mechanisms including phosphorylation, acetylation and adenylation, which are catalyzed by ATP-dependent O-phosphotransferase, actyl-CoA dependent N-acetyltransferase, and ATP-dependent O-nucleotidyl-transferase, respectively^4^. ANT(2″)-Ia and AAC(6′)-II are two important aminoglycosides modifying enzyme in *K. pneumoniae*^5,6^. ANT(2″)-Ia was isolated from *K. pneumoniae* for the first time^7^, and nowadays, has become one of the most important enzymes causing antibiotic resistances in gram-negative pathogens^8–10^. On the other hand, AAC(6′) is the most important and common enzyme in the AAC super-family. More than 40 kinds of this enzyme have been identified. ACC(6′) exists in both gram-negative and gram-positive bacteria^11^.

Integrons play an important role in increasing antibiotic resistance, particularly in gram-negative pathogens. There are five classes of integrons in which class 1 and class 2 are the most common among other classes, and mainly associated with hospital acquired resistance, which often includes resistance to aminoglycosides and beta-lactams, trimethoprim, ampicillin and chloramphenicol^12^. Class 1 integron, which is the main cause of antibiotic resistance spreading, has been studied in many microorganisms. Studies suggest that this integron can be seen in 22–59% of gram-negative rods, especially *K. pneumoniae*^13^.

The number of aminoglycosides resistant *K. pneumoniae* strains is increasing. In addition, genes coding AMEs can be carried by integrons which can be involved in aminoglycosides resistant bacteria. Thus, in this study, the frequency of *aac(6′)-II* and *ant(2″)-Ia* genes and integrons class 1 and 2 were investigated in *K. pneumoniae* isolated from clinical samples of hospitalized patients in Yazd.

## Materials and Methods

### Sample obtaining and culture

In this cross-sectional study, from October 2015 to March 2016, a total of 130 *K. pneumoniae* isolates were obtained from different samples of hospitalized patients in training hospitals of Yazd. The samples were transferred to Microbiology Laboratory of Yazd Shahid Sadoughi University of Medical Sciences, and were cultured in EMB medium (Merck Darmstadt, Germany) and incubated at 37°*C* for 18–24 *hr*.

### Bacteria identification

Lactose-positive colonies were identified by differential biochemical tests including glucose and lactose fermentation in TSI culture medium, indole production and motility on SIM medium, reaction in MR-VP medium, growth on Simmon-citrate medium, and urease production on urea agar (all media from Merck Darmstadt, Germany).

### Antibiotic susceptibility testing

Susceptibility of *K. pneumoniae* isolates to aminoglycoside such as tobramycin (10 *mg*), gentamicin (10 *mg*), amikacin (30 *mg*) and kanamycin (5 *mg*) was performed using disk diffusion method (Kirby-Bauer) (Liofilchem, Italy) recommended by Clinical and Laboratory Standard Institute (CLSI) guideline^14^. In this test, the bacterial suspension with a turbidity which was equal to turbidity of McFarland tube 0.5 (1.5×10^8^ *CFU/ml*) was used. *E. coli* ATCC 25922 was used as the control.

### Measuring the Minimum Inhibitory Concentration (MIC) of gentamicin

E-test method (Liofilcam, Italy) was used to determine the Minimum Inhibitory Concentration (MIC) of gentamicin^15^. Based on CLSI guideline, MIC>16 was considered as resistant isolates to gentamicin^14^. *Escherichia coli (E. coli)* ATCC 25922 was used as control.

### Genomic DNA extraction

Genomic DNA was extracted by salting out method^16^. The quality and quantity of obtained DNA was measured by 0.7% agarose gel electrophoresis and nano-drop, respectively. DNA samples were stored at −20°*C* until the examining day.

### Polymerase Chain Reaction (PCR)

PCR was carried out using thermocycler (Eppendorf, Germany) to evaluate the presence of *ant(2″)-Ia*, *aac(6′)-II*, *intl1*, and *intl2* genes in the isolates. Specific primers were as follows: 5′-GGTGTGGCGGGCTTCGTG-3′ and 5′-GCATCCTCGGTTTTCTGG-3′ as forward and reverse primer for *intl1* (amplicon size: 480 *bp*)^17^; 5′-CTAGAATAGGCTGTATAGGCAGA-3′ and 5′-GAGTGACGAAATGTATGACAAG-3′ as forward and reverse primer for *intl2* (amplicon size: 850 *bp*)^18^; 5′-CACAACGCAGGTCATT-3′ and 5′-CGCTAAGAATCCATAGTCCAA-3′ as forward and reverse primer for *ant(2″)-Ia* (amplicon size: 220 *bp*; designed using Primer3 software); and 5′-GCCGATGCTCCATGATTG-3′ and 5′-TCGAAGGCTTGTCGTGTT-3′ as forward and reverse primer for *aac(6′)-II* (amplicon size: 480 *bp*; designed using Primer3 software).

The final volume for PCR reaction was 20 *ml* (water 5 *μl*), PCR 2X master mix (Amplicon-Denmark) (10 *μl*), working primers with final concentration of 5 *pmol* (2 *μl*), and template DNA (3 *μl*).

The thermal profile was as follows: 94°*C* for 5 *min* as initial denaturation for one cycle; 94°*C* for 60 *s*, 52°*C* for 60 *s*, and 72°*C* for 60 *s*, for 30 cycles; with a final extension at 72°*C* for 5 *min*. The PCR reaction condition was the same for all tested genes, except *intl1* that its annealing temperature was considered 50°*C*. 1% agarose gel electrophoresis was performed on PCR products, and sequenced for further confirmation.

### Statistical analysis

Correlation between the genes and resistance to aminoglycosides were analyzed by chi-square method using SPSS software v. 16.0 (SPSS Inc, Chicago, IL, USA).

## Results

Among all 130 *K. pneumoniae* isolates, aminoglycosides resistance was as follows: 43.8% for kanamycin, 34.6% for gentamicin, 33.8% for tobramycin and 14.6% for amikacin. Generally, 54.6% of isolates were resistant at least to one aminoglycoside. Phenotypic results showed the highest simultaneous resistance of 29.2% for gentamicin-kanamycin, followed by 25.3% for gentamicin-tobramycin, 24.4% for kanamycin-tobramycin, and 9.23% for gentamicin-tobramycin (Table 1).

**Table 1.** Results of antibiotic resistance and studied genes in 130 *K. pneumoniae* isolates

**Antibiotic resistance**	**Resistant n (%)**	***aac(6′)-IIa* gene n (%)**	***ant(2″)-Ia* gene n (%)**	***int1* gene n (%)**
**GM-K-TOB-AK**	12 (9.23)	12 (9.23)	4 (3)	12 (9.23)
**GM-TOB- K**	32 (24.6)	32 (24.6)	13 (10)	32 (24.6)
**GM-AK-K**	14 (10.7)	14 (10.7)	4 (3)	14 (10.7)
**GM-K**	38 (29.2)	38 (29.2)	16 (12.3)	37 (28.4)
**K-TOB**	37 (28.4)	32 (24.6)	16 (12.3)	35 (26.9)
**GM-TOB**	33 (25.3)	33 (25.3)	14 (10.7)	32 (24.6)
**K**	57 (43.8)	45 (34.6)	26 (20)	54 (41.5)
**GM**	45 (34.6)	40 (30)	17 (13)	41 (31.5)
**TOB**	44 (33.8)	34 (26.1)	20 (15.3)	40 (30.7)
**AK**	19 (14.6)	17 (13)	6 (4.6)	17 (13)

K: Kanamycin; TOB: Tobramycin; GM: Gentamicin; AK: Amikacin.

MIC of 45 isolates which were resistant to gentamicin by disc diffusion method was as follows: 37 resistant isolates (83%) and 8 sensitive isolates (18%). MIC was 32 *μg/ml* for 23.9% of isolates (Table 2).

**Table 2.** Results of MIC for gentamicin by E test method

**MIC range (*μg/ml*)**	**2>**	**4**	**8**	**16**	**24**	**32**	**48**	**64**	**256**
**No.**	8	0	0	3	7	11	8	3	5
**Percent**	17.7	0	0	6.6	15.5	24.4	17.7	6.6	11.1

**Figure 1.**
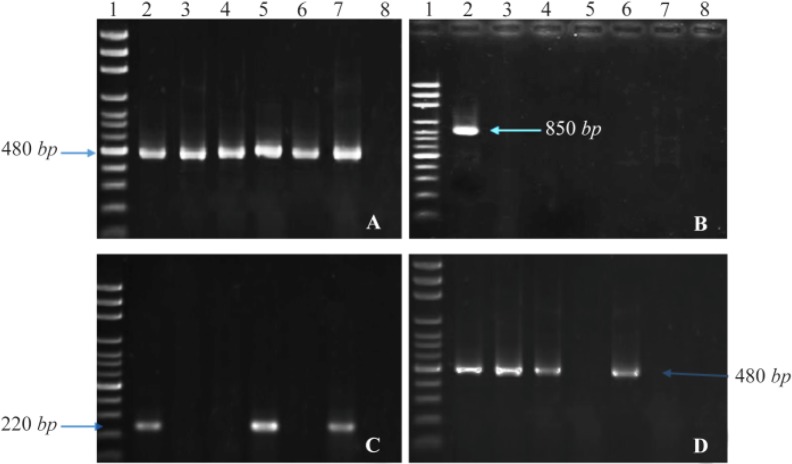
PCR products of *intl1* (A), *intl2* (B), *ant(2″)-Ia* (C), and *aac(6′)-IIa* (D) on agarose gel electrophoresis visualized under UV light. Lane 1 shows DNA Marker; lane 2 shows positive control; lane 3–7 show PCR products of the amplicon amplified from *K. pneumonia* isolates; and lane 8 shows negative control of attributed genes.

Our results showed that frequencies of *aac(6′)-II, ant(2″)-Ia, intl1* in studied isolates were 44.6% (n=58), 27.7% (n=36), and 90% (n=117), respectively. But *intl2* was not found in the isolates (Figure 1). Furthermore, the correlation between presence of these genes and resistance to one particular antibiotic or different mixtures of antibiotics have also been assessed. The results showed that in isolates which were resistant to gentamicin-kanamycin-tobramycin, the presence of *intl1*, *ant(2″)-Ia*, and *aac(6′)-II* was 25.3, 10 and 25.3%, respectively (Table 1). Other correlations are stated in table 1.

## Discussion

*K. pneumoniae* is an opportunistic pathogen of *Enterobacteriaceae* family. *K. pneumoniae* colonization is very common among hospitalized patients. This bacterium causes septicemia, bacteremia, meningitis, urinary and pulmonary tracts infection, and enteritis in children, elderly and patients with immunodeficiency disorders^19^. Aminoglycosides are one the most important treatments used against *K. pneumoniae* infections. Acetylation and adenylation are two important mechanisms of aminoglycosides resistance, catalyzed by actyl-CoA dependent N-acetyltransferase, and ATP-dependent O-*nucleotidyltransferase*, respectively^4^. In this study, resistance to aminoglycosides and the frequencies of *aac (6′)-IIa* and *ant(2″)-Ia* genes which encode the most important acetyl- and adenyl-transferases in nosocomial pathogens^4^, have been evaluated in *K. pneumoniae* isolates. Moreover, since integrons specially class 1 integron are one of the main causes of antibiotic resistance among bacterial populations^13,20^, also the frequencies of *intl1* and *intl2* genes were evaluated in *K. pneumoniae* isolates.

Our results showed that resistance of *K. pneumoniae* isolates to gentamicin, kanamycin, tobramycin, and amikacin was 34.6, 43.8, 33.8, and 14.6%, respectively. Moreover, according to E test results, most isolates (24.4%) had MIC at range of 32 for gentamicin. Najar-Peerayeh *et al* who studied 200 *K. pneumoniae* isolates revealed that the frequency of resistance to gentamicin, tobramycin, and amikacin were 36, 32, and 27%, respectively^21^. Our results about resistance to gentamicin and tobramycin were consistent with theirs, but percentage of resistance to amikacin was nearly doubled in their study (27 *vs*. 14.6%). Study of Mobarak-Qamsari *et al* on 104 *K. pneumoniae* isolates revealed that the frequency of resistance for kanamycin, tobramycin, gentamicin, and amikacin was 48, 28.6, 25.9, and 25.9%, respectively^22^. These results are nearly consistent with ours, except resistance percentage to amikacin, which was doubled in their study. In fact, these two studies that carried out in Tehran (Iran) showed same results about amikacin. The observed differences might be due to the geographical differences, and wide prescription of antibiotics in that area. However, Li *et al* showed that frequency of resistance to gentamicin, tobramycin, and amikacin in China, was 62.2, 71.1, and 31.4%, respectively. These results are very different from the results obtained from Iran. These results showed that frequency of resistance to aminoglycosides could vary in different countries, and even hospitals.

The relationship between *aac(6′)-II* and *ant(2″)-I*, two important genes coding aminoglycosides modifying enzymes, with resistance to the aminoglycosides demonstrates that *aac(6′)-II* gene is significantly associated with resistance to these four antibiotics (p= 0.000). 93.3% of resistant isolates were positive to *aac(6′)-II* gene, while only 18.82% of susceptible isolates contained this gene. Among kanamycin resistant isolates, 77.19% of them were *aac(6′)-II* gene positive, but 17.14% of susceptible isolates possess this gene. Moreover, this gene was present in amikacin and tobramycin resistant isolates at a frequency of 94.73 and 81.81%, respectively, while in susceptible isolates by 33.01, and 22.22% respectively. In the present study, *aac(6′)-II* gene was found in 44.6% of isolates. Interestingly, in Najar-Peerayeh *et al*^21^ study, this gene was found in 42.5% of isolates. Moreover, Liang *et al*^23^ reported that 30.25% of the isolates were positive for this gene. Thus, our results are consistent with these studies.

*ant(2″)-I* gene was found in gentamicin resistant and gentamicin susceptible isolates at the rate of 34.77, and 22.35%, respectively. The relationship between this gene and gentamicin resistance was not statically significant (p=0.068). But, there was a significant association between this gene with resistance to kanamycin (p=0.001), and 43.85% of the kanamycin resistant isolates had this gene, while just 15.71% of the susceptible ones had it. Our results also showed that there is a significant correlation between *ant(2″)-I* and resistance to tobramycin (p=0.003)(data in details are not shown here). The results showed that 47.72% of resistant isolates and 16.04% of susceptible isolates had this gene. Based on our study, there was no significant correlation between *ant(2″)-I* gene and resistance to amikacin (p=0.31) (data in details are not shown here). Generally, *ant(2″)-I* gene was found in 27.7% of the studied isolates. The presence of this gene was reported 13.59% in Liang *et al’s*^23^ study.

In the next section of the present study, the frequencies of *intl1* and *intl2* genes were evaluated. PCR results showed that 90% (n=117) of isolates had *intl1* gene; but *intl2* was not found in the studied isolates. Integron class 1 is considered as an important genetic element carrying resistance genes to antibiotics. Keeping this in mind, the correlation of *intl1* gene with the mentioned resistance, and with *ant(2″)-Ia* and *aac(6′)-II* genes was evaluated. High frequency of *intl1* gene was found in resistant isolates. The frequency of this gene in resistant isolates to gentamicin, kanamycin, amikacin, and tobramycin was 93.3, 94.73, 89.47, and 93.18%, respectively; on the other hand in susceptible isolates, the frequencies were 88.23, 85.71, 90.56, and 87.65%, respectively. There was no significant correlation between this gene and the mentioned resistance (p>0.05). Moreover, there was no significant association between *intl1* gene and *aac(6′)-II* and *ant(2″)-I* genes (p=0.29) (data in details are not shown here).

The frequency of integron class 1 was reported 51.1% in the study of Li *et al*^24^. They showed that there is a significant correlation between *intl1* and resistance to antibiotics. Despite high frequency of class 1 integron in our study, no significant correlation between *intl1* and resistance to antibiotics was observed. Comparing our results with the study of Li *et al*, high frequency of *intl1* gene in the studied isolates was found. These reports are really alarming, because class 1 integron is one of the most important genetic elements that cause expansion of antibiotic resistance in hospitals, and acquiring resistance to drugs. Given the high frequency of class 1 integron in this study and its effect on increasing resistance to antibiotics, especially aminoglycosides, it can be concluded that more attention is needed in this area because they can easily create resistance to the antibiotics, and reduce the sensitivities of isolates to aminoglycosides. Since class I interon was found in many susceptible isolates of this study, and given the fact that specific antibiotic resistance genes are carried by gene cassettes in integrons^20^, the correlation of cassettes and genes encoding resistance to antibiotics should also be examined, to figure out the association between *intl1* gene and resistance to the aminoglycosides. Considering that *intl2* gene was not found in these *K. pneumoniae* isolates, further studies are required.

## Conclusion

Our results showed high frequencies of *aac(6′)-IIa*, *ant(2″)-Ia*, and *intl1* genes in *K. pneumoniae* isolates which could be the results of unnecessary prescription of antibiotics. These results revealed the importance of antibiotic resistance gene. Moreover, selecting an antibiotic by antibiotic susceptibility testing has an important role in treatment and inhibiting the resistance to antibiotics. Since aminoglycosides are very important in treatment of *K. pneumoniae* infections, thus, the prevalence rate of this resistance should be evaluated annually in countries.
